# Natural Dibenzo-α-Pyrones and Their Bioactivities

**DOI:** 10.3390/molecules19045088

**Published:** 2014-04-22

**Authors:** Ziling Mao, Weibo Sun, Linyun Fu, Haiyu Luo, Daowan Lai, Ligang Zhou

**Affiliations:** MOA Key Laboratory of Plant Pathology, Department of Plant Pathology, College of Agronomy and Biotechnology, China Agricultural University, Beijing 100193, China

**Keywords:** dibenzo-α-pyrones, dibenzo-α-pyranones, 6*H*-benzo[*c*]chromen-6-ones, 6*H*-dibenzo[*b*,*d*]pyran-6-ones, biological activities

## Abstract

Natural dibenzo-α-pyrones are an important group of metabolites derived from fungi, mycobionts, plants and animal feces. They exhibit a variety of biological activities such as toxicity on human and animals, phytotoxicity as well as cytotoxic, antioxidant, antiallergic, antimicrobial, antinematodal, and acetylcholinesterase inhibitory properties. Dibenzo-α-pyrones are biosynthesized via the polyketide pathway in microorganisms or metabolized from plant-derived ellagitannins and ellagic acid by intestinal bacteria. At least 53 dibenzo-α-pyrones have been reported in the past few decades. This mini-review aims to briefly summarize the occurrence, biosynthesis, biotransformation, as well as their biological activities and functions. Some considerations related to synthesis, production and applications of dibenzo-α-pyrones are also discussed.

## 1. Introduction

**Figure 1:**
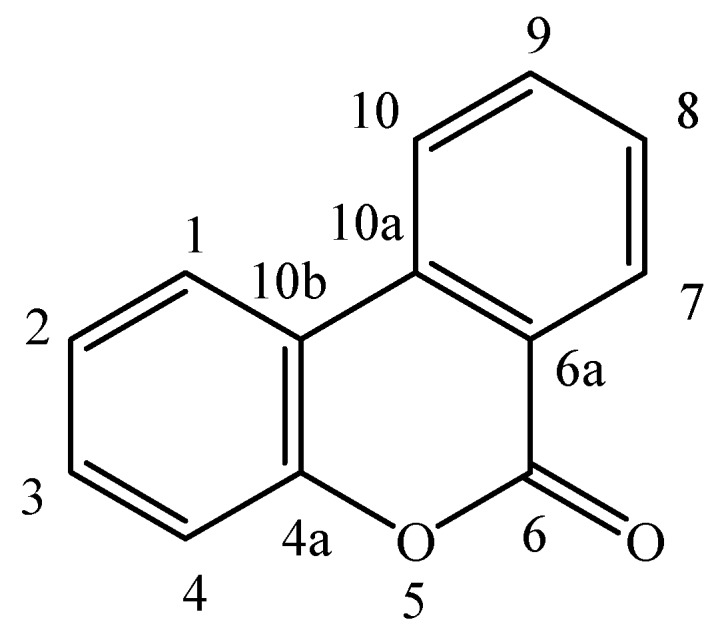
The basic skeleton of dibenzo-α-pyrones.

Dibenzo-α-pyrones (also named dibenzo-α-pyranones, 6*H*-benzo[*c*]chromen-6-ones, and 6*H*-dibenzo[*b*,*d*]pyran-6-ones) are an important group of heptaketide coumarin derivatives that have a fused tricyclic nucleus (Figure 1). They are usually isolated from fungi [1], mycobionts [2,3], plants [4,5], and animal feces containing the transformed products of plant-derived ellagitannins and ellagic acid by intestinal bacteria [6,7]. Many of them possess a wide spectrum of biological activities, spanning from toxicity on human and animals [8], cytotoxic activity [9], phytoxicity [10], antioxidant [6], antiallergic [11], antimicrobial [12], to acetylcholinesterase inhibitory activities [13]. In addition, dibenzo-α-pyrones are key intermediates in the synthesis of cannabinoids [14], and other pharmaceutically interesting compounds such as progesterone, androgen, glucocorticoid receptor agonists [15,16], as well as endothelial proliferation inhibitors [17], and antidyslipidemic agents [18]. This review mainly presents the occurrence, biosynthesis, biotransformation, and biological activities of the dibenzo-α-pyrones from bioorganisms. We also discuss and prospect their synthesis, production and applications.

## 2. Occurrence

### 2.1. Dibenzo-α-pyrones from Fungi

**Figure 2:**
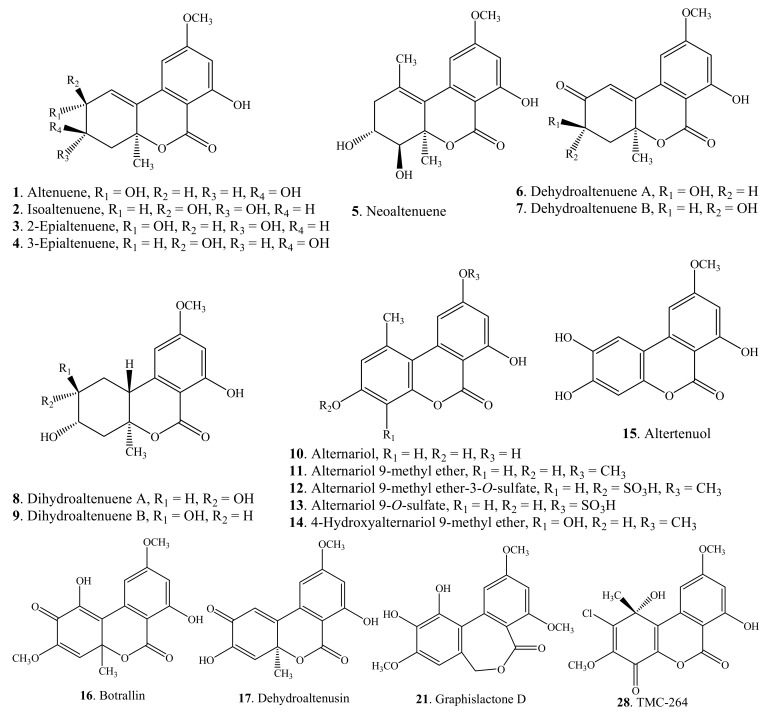

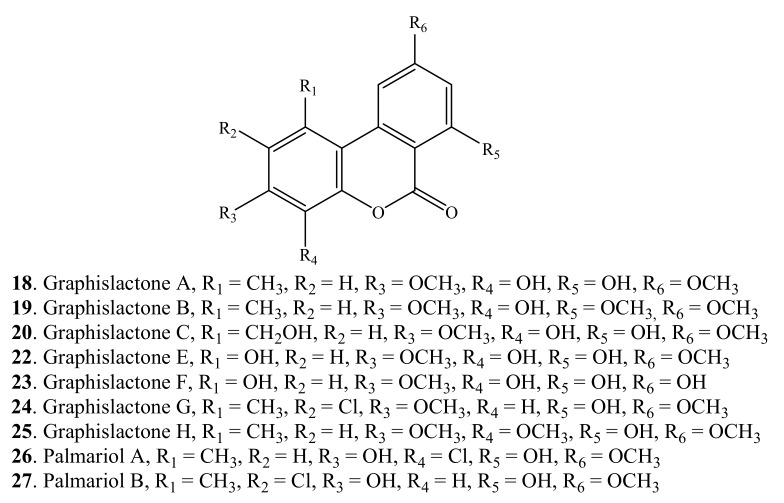
Structures of dibenzo-α-pyrones isolated from fungi.

Dibenzo-α-pyrones are mainly distributed in the *Alternaria* species and mycobionts. Other dibenzo-α-pyrone-producing fungi include *Botrytis allii*, *Cephalosporium acremonium*, *Hyalodendriella* sp. Ponipodef12, *Microsphaeropsis olivacea*, *Penicillium verruculosum*, and *Phoma* sp. TC 1674 (Table 1). From the biosynthetic pathway, fungal dibenzo-α-pyrones have a polyketide origin via acetyl-CoA and malonyl-CoA. They are usually toxic to plants and animals. Typical examples include alternariol (**10**), alternariol 9-methyl ether (**11**), botrallin (**16**), and 2-chloro-4,6-dihydro-1,7-dihydroxy-3,9-dimethoxy-1-methyl-1*H*-dibenzo[*b,d*]pyran-4,6-dione (TMC-264, **28**). The structures of the dibenzo-α-pyrones from fungi are shown in Figure 2.

**Table 1:** Occurrence of the dibenzo-α-pyrones in fungi.

Dibenzo-α-pyrone	Fungal Species	Reference
Altenuene = ATL (**1**)	Endophytic *Alternaria* sp. isolated from *Polygonum senegalense*	[9]
	*Alternaria alternata*	[10]
	Unidentified freshwater fungus belong to Tubeufiaceae	[19]
Isoaltenuene (**2**)	*Alternaria alternata*	[20]
	Unidentified freshwater fungus belong to Tubeufiaceae	[19]
2-Epialtenuene (**3**)	*Alternaria alternata*	[21]
	Unidentified freshwater fungus belong to Tubeufiaceae	[19]
3-Epialtenuene (**4**)	Endophytic *Alternaria* sp. isolated from *Polygonum senegalense*	[9]
Neoaltenuene (**5**)	*Alternaria alternata*	[21]
Dehydroaltenuene A (**6**)	Unidentified freshwater fungus belong to Tubeufiaceae	[19]
Dehydroaltenuene B (**7**)	Unidentified freshwater fungus belong to Tubeufiaceae	[19]
Dihydroaltenuene A (**8**)	Unidentified freshwater fungus belong to Tubeufiaceae	[19]
Dihydroaltenuene B (**9**)	Unidentified freshwater fungus belong to Tubeufiaceae	[19]
Alternariol = AOH (**10**)	Endophytic *Acremonium* sp. isolated from *Plantago lanceolata*	[22]
	Endophytic *Alternaria* sp. isolated from *Polygonum senegalense*	[9]
	Endophytic *Alternaria* no.28 isolated from *Ginkgo biloba*	[23]
	Endophytic *Alternaria* sp. PR-14 isolated from *Paeonia delavayi*	[24]
	Endophytic *Alternaria* sp. isolated from *Datura stramonium*	[25]
	Endophytic *Alternaria* sp. N.SBA10 isolated from *Scutellaria baicalensis*	[26]
	*Alternaria alternata*	[27]
	*Alternaria alternata*	[10]
	Endophytic *Alternaria brassicicola* ML-P08 isolated from *Malus halliana*	[28]
	Endophytic *Alternaria tenuissima* EN-192 isolated from *Rhizophora stylosa*	[29]
	Endophytic *Colletotrichum* sp. isolated from *Aristolochia* sp.	[30]
Alternariol 9-methyl ether = AME = Djalonensone (**11**)	Endophytic *Acremonium*sp. isolated from *Plantago lanceolata*	[22]
	Endophytic *Alternaria* sp. isolated from *Polygonum senegalense*	[9]
	Endophytic *Alternaria* sp. PR-14 isolated from *Paeonia delavayi*	[24]
	Endophytic *Alternaria* sp. isolated from *Datura stramonium*	[25]
	Endophytic *Alternaria* sp. N.SBA10 isolated from *Scutellaria baicalensis*	[26]
	*Alternaria alternata*	[27]
	*Alternaria alternata*	[31]
	*Alternaria alternata*	[10]
	Endophytic *Alternaria* no.28 isolated from *Ginkgo biloba*	[23]
	Endophytic *Alternaria brassicicola* ML-P08 isolated from *Malus halliana*	[28]
	Endophytic *Alternaria linicola* isolated from *Linum ustiatissimum*	[32]
	*Alternaria tenuis*	[33]
Alternariol 9-methyl ether = AME = Djalonensone (**11**)	Endophytic *Alternaria tenuissima* isolated from *Acacia mangium*	[34]
	Endophytic *Alternaria tenuissima* EN-192 isolated from *Rhizophora stylosa*	[29]
	Endophytic *Cephalosporium acremonium* IFB-E007 isolated from *Trachelospermum jasminoides*	[35]
	Endophytic *Colletotrhichum* sp. isolated from *Aristolochia* sp.	[30]
	Endophytic *Hyalodendriella* sp. Ponipodef12 isolated from the hybrid ‘Neva’ of *Populus deltoides × P. nigra*	[12]
	*Lachmum palmae*	[36]
Alternariol 9-methyl ether-3-*O*-sulfate (**12**)	Endophytic *Alternaria* sp. isolated from *Polygonum senegalense*	[9]
Alternariol 9-*O*-sulfate (**13**)	Endophytic *Alternaria* sp. isolated from *Polygonum senegalense*	[9]
4-Hydroxyalternariol 9-methyl ether (**14**)	Endophytic *Alternaria* sp. isolated from *Polygonum senegalense*	[9]
	Endophytic *Alternaria* sp. isolated from *Datura stramonium*	[25]
Altertenuol = Altenuisol = Alternuisol (**15**)	*Alternaria* sp.	[37]
	*Alternaris tenuis*	[38]
	*Alternaris tenuis*	[39,40]
	*Alternaris tenuis*	[41]
Botrallin (**16**)	*Botrytis allii*	[42]
	Endophytic *Hyalodendriella* sp. Ponipodef12 isolated from the hybrid ‘Neva’ of *Populus deltoides × P. nigra*	[12,43]
	Endophytic *Microsphaeropsis olivacea* isolated from *Pilgerodendron uviferum*	[13]
Dehydroaltenusin (**17**)	*Acremonium* sp.	[44]
	*Alternaria tenuis*	[45]
	*Penicillium verruculosum*	[46]
Graphislactone A (**18**)	Mycobiont of *Graphis scripta* var. *pulverulenta*	[2]
	Endophytic *Cephalosporium acremonium* IFB-E007 isolated from *Trachelospermum jasminoides*	[35]
	Endophytic *Microsphaeropsis olivacea* isolated from *Pilgerodendron uviferum*	[13]
Graphislactone B (**19**)	Mycobiont of *Graphis scripta* var. *pulverulenta*	[2]
Graphislactone C (**20**)	Mycobiont of *Graphis scripta* var. *pulverulenta*	[2]
Graphislactone D (**21**)	Mycobiont of *Graphis scripta* var. *pulverulenta*	[2]
Graphislactone E (**22**)	Mycobiont of *Graphis scripta*	[3]
	Mycobiont of *Graphis prunicola*	[3]
Graphislactone F (**23**)	Mycobiont of *Graphis prunicola*	[3]
Graphislactone G (**24**)	Endophytic *Cephalosporium acremonium* IFB-E007 isolated from *Trachelospermum jasminoides*	[35]
Graphislactone H (**25**)	Endophytic *Cephalosporium acremonium* IFB-E007 isolated from *Trachelospermum jasminoides*	[35]
Palamriol A (**26**)	*Lachmum palmae*	[36]
Palmariol B (**27**)	*Lachmum palmae*	[36]
	Endophytic *Hyalodendriella* sp. Ponipodef12 isolated from the hybrid ‘Neva’ of *Populus deltoides × P. nigra*	[12]
TMC-264 (**28**)	*Phoma* sp. TC 1674	[47]

### 2.2. Dibenzo-α-pyrones from Plants

The dibenzo-α-pyrones from plants are listed in Table 2. One dibenzo-α-pyrone, namely djalonensone (**11**), was isolated from the roots of *Anthocleista djalonensis* (Loganiaceae). The authors postulated djalonensone to be a significant taxonomic marker of the plant species [48]. However, djalonensone is identical to alternariol 9-methyl ether (AME) which has been isolated from a series of fungi including pathogenic and endophytic fungi [1]. Thus, the significance of djalonensone (**11**) as an important taxonomic marker of the plant species should be reconsidered. The possibility that djalonensone (**11**) was produced by an endophytic fungus residing in the healthy roots of *A. djalonensis*, needs further investigation [49].

**Figure 3:**
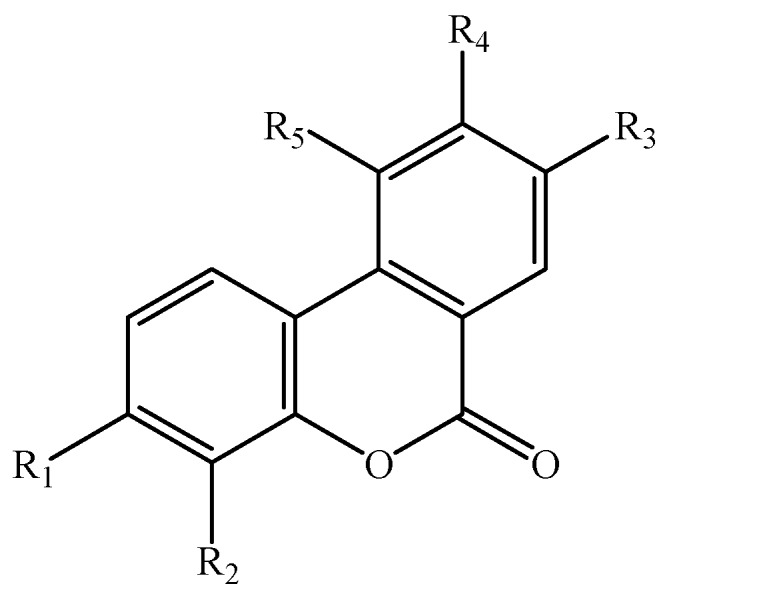
Dibenzo-α-pyrones produced by transformation of intestinal bacteria.

**Figure 4:**
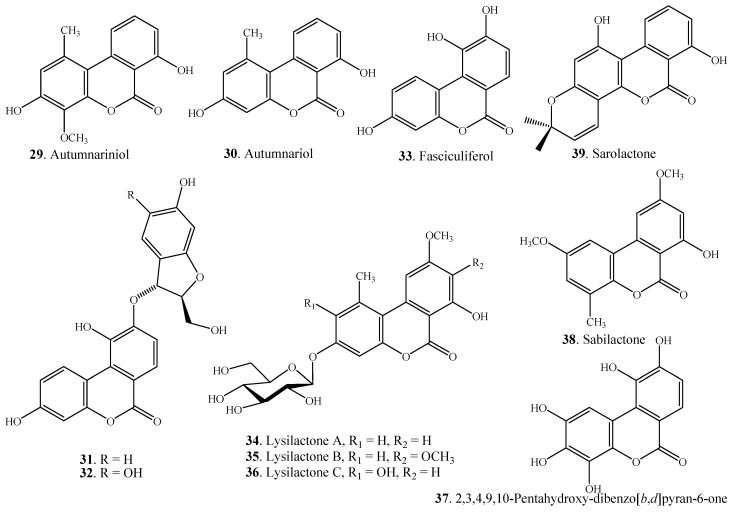
Structures of the dibenzo-α-pyrones from plants.

Three dibenzo-α-pyrones, namely (2'*S*,3'*R*)-3,10-dihydroxy-9-*O*-(6'-hydroxy-2'-hydroxymethyl- dihydrofuran-3-yl)-dibenzo[*b*,*d*]pyran-6-one (**31**), (2'*S*,3'*R*)-3,10-dihydroxy-9-*O*-(5',6'-dihydroxy-2'-hydroxymethyldihydrofuran-3-yl)-dibenzo[*b*,*d*]pyran-6-one (**32**), and fasciculiferol (**33**) were isolated from the heartwood of *Umtiza listerana* [50]. Fasciculiferol (**33**) was previously isolated from the heartwood of *Acacia fasciculifera* [51]. Two dibenzo-α-pyrones, autumnariniol (**29**) and autumnariol (**30**), were isolated from the bulbs of *Eucomis autumnalis* (Liliaceae) [52]. Four urolithins, namely urolithin A (**40**), isourolithin A (**41**), urolithin B (**42**) and urolithin C (**43**) were isolated from *Trapa natans* (Trapaceae) [4] and *Caesalpinia sappan* (Caesalpiniaceae) [53]. These urolithins (Figure 3) were also isolated from animal feces [6,7]. Other dibenzo-α-pyrones isolated from plants included sabilactone (**38**) from *Sabina vulgaris* [54] and sarolactone (**39**) from *Hypericum japonicum* [55]. The structures of the dibenzo-α-pyrones from plants are shown in Figure 4.

**Table 2:** Occurrence of dibenzo-α-pyrones in plants.

Dibenzo-α-pyrone	Plant species (Family)	Reference
Alternariol 9-methyl ether (**11**)	*Anthocleista djalonensis* (Loganiaceae)	[48]
Autumnariniol (**29**)	*Eucomis autumnalis* Graeb (Liliaceae)	[52]
Autumnariol (**30**)	*Eucomis autumnalis* Graeb (Liliaceae)	[52]
(2' *S*,3'*R*)-3,10-Dihydroxy-9-*O*-(6'- hydroxy-2'-hydroxymethyldihydrofuran-3-yl)-dibenzo[*b*,*d*]pyran-6-one (**31**)	*Umtiza listerana* (Caesalpiniaceae)	[50]
(2' *S*,3'*R*)-3,10-Dihydroxy-9-*O*-(5',6'-dihydroxy-2'-hydroxymethyldihydrofuran-3-yl)-dibenzo[*b*,*d*]pyran-6-one (**32**)	*Umtiza listerana* (Caesalpiniaceae)	[50]
Fasciculiferol (**33**)	*Acacia fasciculifera* (Mimosaceae)	[51]
	*Umtiza listerana* (Caesalpiniaceae)	[50]
Lysilactone A (**34**)	*Lysimachia clethroides* (Primulaceae)	[5]
Lysilactone B (**35**)	*Lysimachia clethroides* (Primulaceae)	[5]
Lysilactone C (**36**)	*Lysimachia clethroides* (Primulaceae)	[5]
2,3,4,9,10-Pentahydroxy-6 *H*-dibenzo[*b*,*d*]pyran-6-one (**37**)	*Chrozophora senegalensis* (Euphorbiaceae)	[56]
	*Polygonum chinense* (Polygonaceae)	[57]
	*Sebastiania chamaelea* (Euphorbiaceae)	[56]
	*Tamarix nilotica* (Tamaricaceae)	[58]
Sabilactone (**38**)	*Sabina vulgaris* (Cupressaceae)	[54]
Sarolactone (**39**)	*Hypericum japonicum* (Guttiferae)	[55]
Urolithin A (**40**)	*Trapa natans* (Trapaceae)	[4]
Isourolithin A (**41**)	*Trapa natans* (Trapaceae)	[4]
Urolithin B (**42**)	*Trapa natans* (Trapaceae)	[4]
Urolithin C (**43**)	*Caesalpinia sappan* (Caesalpiniaceae)	[53]

**Table:** 

Dibenzo-α-pyrone	R_1_	R_2_	R_3_	R_4_	R_5_	Reference
Urolithin A (**40**)	OH	H	OH	H	H	[6]
Urolithin B (**42**)	OH	H	H	H	H	[6]
Urolithin C (**43**)	OH	H	OH	OH	H	[6]
Urolithin D (**44**)	OH	OH	OH	OH	H	[59]
Urolithin E (**45**)	OH	OH	OH	H	OH	[59]
Urolithin M-5 (**46**)	OH	OH	OH	OH	OH	[7]
Urolithin M-6 (**47**)	OH	H	OH	OH	OH	[7,59]
Urolithin M-7 (**48**)	OH	H	OH	H	OH	[7,11]
Isourolithin A (**41**)	OH	H	H	OH	H	[7]
Isourolithin B (**49**)	H	H	H	OH	H	[7]
8-*O*-Methylurolithin A (**50**)	OH	H	OCH_3_	H	H	[6]
8,9-Di-*O*-methylurolithin C (**51**)	OH	H	OCH_3_	OCH_3_	H	[6]
8,9-Di-*O*-methylurolithin D (**52**)	OH	OH	OCH_3_	OCH_3_	H	[6]

### 2.3. Dibenzo-α-pyrones Produced by Transformation of Intestinal Bacteria

A group of dibenzo-α-pyrones **40**–**52**, namely urolithins with different phenolic hydroxylation patterns, have been isolated from animal feces. Ellagitannins and ellagic acid (EA) are plant secondary metabolites that have relevant antioxidant activities *in vitro*, potential cardiovascular protection, anticarcinogenic and anti-inflammatory effects [59,60,61]. These dibenzo-α-pyrones are important constituents in different foods including pomegranates, berries (*i.e*., strawberry, raspberry, blackberry, and camu-camu), nuts (*i.e*., walnuts, acorns, and chestnuts), muscadine grapes, oak-aged wines, medicinal plants and tisanes (*i.e*., geranium and oak leaves). They are not absorbed in the gut and are metabolized *in vivo* by the intestinal bacteria to produce a series of metabolites known as urolithins [62,63]. Some urolithins such as urolithins A (**40**), B (**42**) and C (**43**) as well as isourolithin A (**41**) were previously isolated from plants (Table 2) [4,53]. The structures of the isolated urolithins are shown in Figure 3.

### 2.4. Dibenzo-α-pyrones from Bacteria

**Figure 5:**
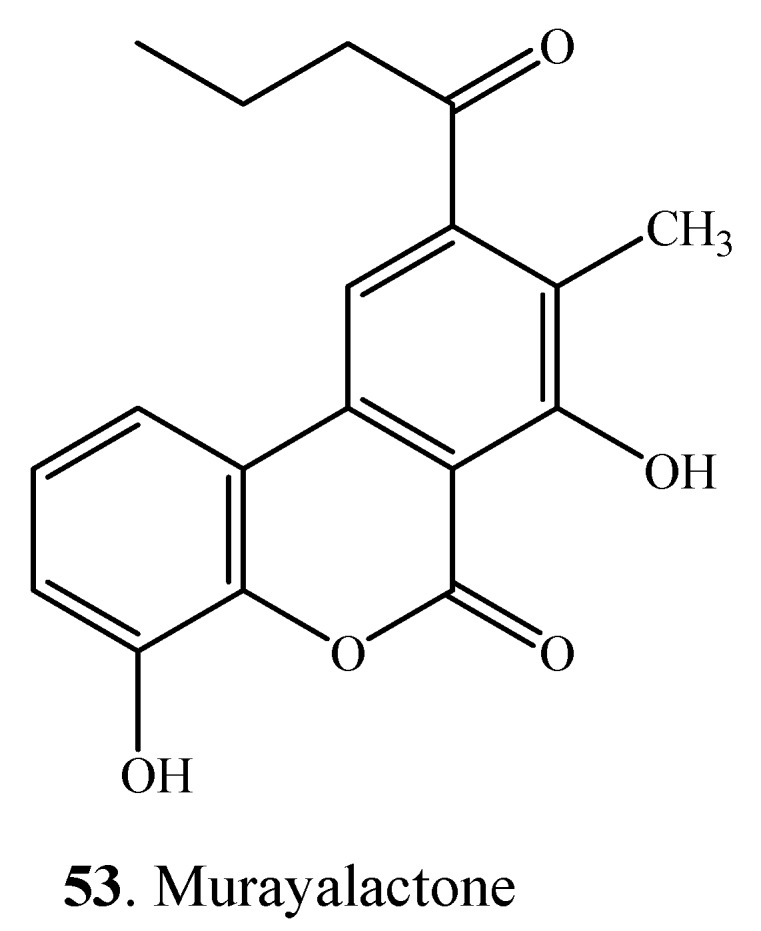
The structure of murayalactone (**53**).

Up to now, only one dibenzo-α-pyrone called murayalactone (**53**) has been isolated from *Streptomyces murayamaensis* [64]. The structure of murayalactone is shown in Figure 5.

## 3. Biosynthesis and Biotransformation

**Scheme 1:**
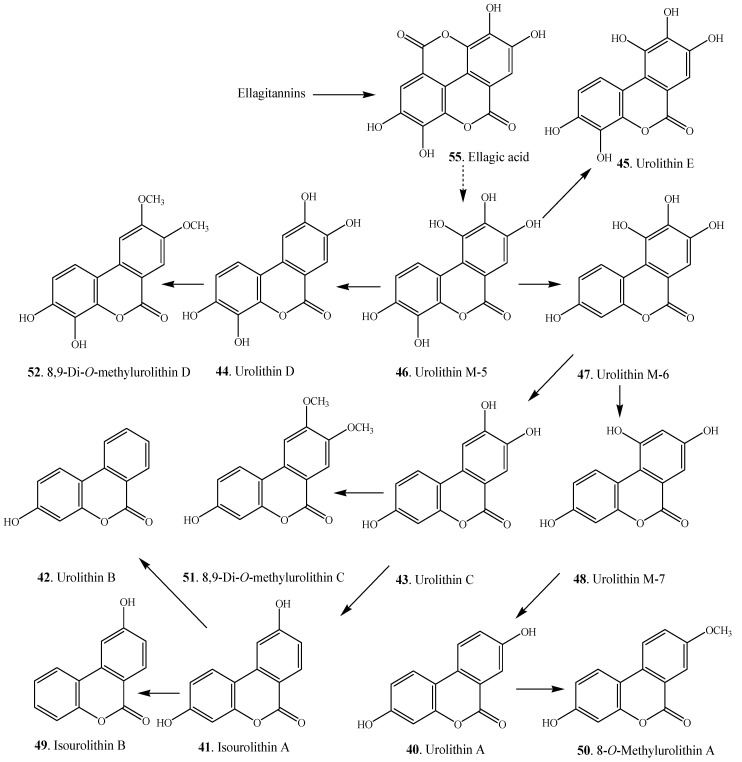
Proposed transformation from ellagic acid to urolithins by intestinal bacteria [6,7,63].

We know very little about the biosynthesis of dibenzo-α-pyrones in living organisms, including their genetics, biochemistry and biosynthetic pathways [2,25,65,66]. In plants, gallic acid (**54**), which is biosynthesized via the shikimate pathway [67], was considered as the precursor of ellagitannins [68]. The ellagitannins would be transformed into ellagic acid (**55**), and then into a series of urolithins (Scheme 1) [6,7,63]. However, in microorganisms, dibenzo-α-pyrones are biosynthesized via the polyketide pathway [3,25]. Polyketide synthase (PKS) is one of the postulated core enzymes in the biosynthesis of 6*H*-dibenzo[*b*,*d*]pyran-6-ones (*i.e*., alternariol, AME) in *Alternaria alternata* [66]. In a draft genome sequence of *A. alternata*, 10 putative PKS-encoding genes were identified. The timing of the expression of two PKS genes, *pksJ* and *pksH*, was found to correlate with the production of AOH and AME [66].

**Scheme 2:**
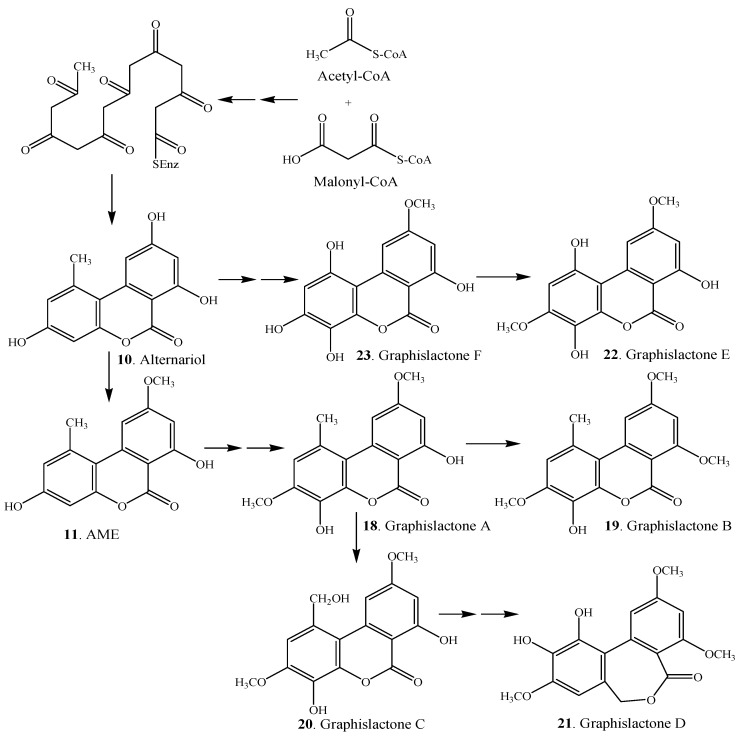
Biosynthetic pathways of graphislactones in the cultured lichen mycobionts [3].

Alternariol (**10**) was first thought to be biosynthesized via norlichexanthone [65], which was ruled out later, and it was proven that alternariol (**10**) could be biosynthesized by simple cyclization and aromatization of a polyketide precursor [69]. After administration of [1-^13^C]acetate and [1,2-^13^C]acetate to cultured lichen mycobionts of *Graphis* spp., acetate units were incorporated into the 6*H*-dibenzo[*b*,*d*]pyran-6-one derivatives including alternariol (**10**), AME (**11**) and graphislactones A-F (**18**–**23**) [3]. Alternariol (**10**) could suffer oxidative demethylation at C-1, hydroxylation at C-4, and *O*-methylation at C-9 to lead formation of graphislactones E (**22**) and F (**23**). On the other hand, alternariol (**10**) could be transformed to graphislactones A-D (**18**–**21**) without demethylation via AME (**11**) [3]. The biosynthetic pathways of graphislactones A-F (**18**–**23**) in the cultured lichen mycobionts of *Graphis* spp. are shown in Scheme 2.

**Scheme 3:**
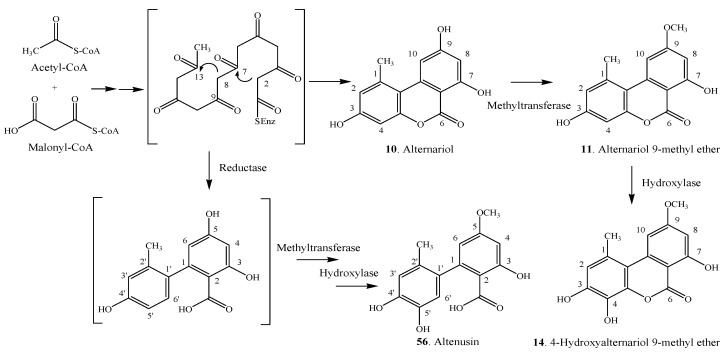
Hypothetical biosynthetic pathways of alternariol (**10**) and its derivatives (**11**, **14**) in an endophytic fungus from *Datura stramonium* [25].

Epigenetic modifiers, including DNA methyltransferase (DNMT) inhibitors (*i.e*., 5-azacytidine, abbreviated as 5-AC) and histone deacetylase (HDAC) inhibitors (*i.e*., suberoylanilide hydroxamic acid, abbreviated as SBHA) are useful to induce the expression of otherwise silent biosynthetic genes under standard laboratory conditions [70]. Supplementation of a DNMT inhibitor 5-AC or a HDAC inhibitor SBHA to the medium induced the production of alternariol (**10**), alternariol 9-methyl ether (**11**), 4-hydroxyalternariol 9-methyl ether (**14**) and altenusin (**56**) [25]. The proposed biosynthesis of the aromatic polyketides **10**, **11**, **14** and **56** involves the condensation of seven molecules of malonyl-CoA, followed by aldol-type cyclizations between C-2 and C-7, and C-8 and C-13, and the subsequent lactonization leads to alternariol (**10**) (Scheme 3). On the other hand, subsequent methylation of the C-9 hydroxyl group of alternariol (**10**) by a methyltransferase results in 4-hydroxyalternariol 9-methyl ether (**14**). Furthermore, the reduction of the C-9 carbonyl group of the heptaketide intermediate by a reductase, and subsequent aldol-type cyclization would produce a biphenyl. Methylation of the C-5 hydroxyl group, and the hydroxylation of C-5' would then lead to altenusin (**56**). The hypothetical biosynthetic pathways [25] of alternariol (**10**) and its derivatives **11**, **14** in an endophytic fungus from *Datura stramonium* are shown in Scheme 3.

Urolithins include a family of metabolites of dibenzo-α-pyrone structures with different phenolic hydroxylation patterns. They are produced in different animals after the intake of ellagitannins and ellagic acid (EA) [71,72]. Ellagitannins are hydrolyzed to ellagic acid (**55**) in the acidic environment of the stomach by the action of the intestinal bacteria. The proposed transformation from ellagic acid to urolithins by the intestinal bacteria [6,7,63] is shown in Scheme 1.

## 4. Biological Activities and Functions

Dibenzo-α-pyrones and their derivatives with diverse chemical properties have been clarified (Figure 1, Figure 2, Figure 3, Figure 4 and Figure 5, Table 1 and Table 2). Some of them act as mycotoxins to humans and animals or as phytotoxins to plants. They have been examined to have a variety of biological activities and functions, which mainly include the cytotoxic, antioxidant, antiallergic, antimicrobial, antinematodal, and acetyl-cholinesterase inhibitory activities.

### 4.1. Toxicity on Human and Animals

The association of mycotoxins from *Alternaria* fungi with human and animal health is not a recent phenomenon. *Alternaria* toxins have been linked to a variety of adverse effects (*i.e.*, genotoxic, mutagenic, and carcinogenic) on human and animal health [8]. Altenuene (**1**), alternariol (**10**), and alternariol 9-methyl ether (**11**) were studied for their toxicity to chickens. Addition of these compounds in chicken feed from sublethal to lethal levels progressively reduced feed efficiency, suppressed weight gain and increased internal haemorrhaging [27,73]. 

There were a few reports about the toxicity of *Alternaria* metabolites on brine shrimp (*Artemia salina* L.) [74,75]. The LC_50_ values of altenuene (**1**) and alternariol (**10**) were 375 and 100 µg/mL, respectively, to brine shrimp larvae by using the disk method of inoculation and an exposure period of 18 h [75]. Altenuene (**1**) and alternariol (**10**) along with alternariol 9-methyl ether (**11**) were also verified to be toxic to brine shrimp [74].

### 4.2. Cytotoxic Activity

Among *Alternaria* dibenzo-α-pyrones, alternariol (**10**) was the most active metabolite to have cytotoxic activity on L5178Y mouse lymphoma cells [9], as well as to have inhibitory activity on protein kinase and xanthine oxidase [28]. Further investigation showed that alternariol (**10**) was a topoisomerase I and II poison which might contribute to the impairment of DNA integrity in human colon carcinoma cells [73,76]. It induced cell death by activation of the mitochondrial pathway of apoptosis in human colon carcinoma cells [76]. Alternariol (**10**) and its 9-methyl ether (**11**) induced cytochrome P450 1A1 and apoptosis in murine heptatoma cells dependent on the aryl hydrocarbon receptor [77]. Other alternariol derivatives such as alternariol 9-methyl ether (**11**), alternariol 9-*O*-sulfate (**13**), and altenusin (**56**) were also screened to be cytotoxic [9].

Dehydroaltenusin (**17**), isolated from *A. tenuis*, was found to be a specific inhibitor of eukaryotic DNA polymerase α to show its strong cytotoxic activity on tumor cells [45,78]. This compound also exhibited strong inhibitory activity on mammalian DNA polymerase α *in vitro* [79]. It was further proved to abrogate cell proliferation of the cultured mammalian cells to show its potential as an effective chemotherapeutic agent against tumors [44].

Alternariol 9-methyl ether (**11**), graphislactone A (**18**), graphislactone G (**24**), and graphislactone H (**25**) from the endophytic fungus *Cephalosporium acremonium* IFB-E007 derived from *Trachelospermum jasminoides* showed pronounced activity against SW1116 cell with IC_50_ values of 14.0, 21.0, 12.0 and 8.5 μg/mL, respectively [35].

Urolithins derived from ellagic acid (**55**) were screened to have cytotoxic and anti-tumor activities. Urolithin A (**40**) inhibited cell growth of human colon cancer cell lines HT29 by inhibiting the canonical wnt signal pathway and interfere with β-catenin/TCF-dependent transcription [80], and inhibited growth of 22Rv1 prostate cancer cells by interfered with the expression of CYP1B1 protein [81]. Urolithin A (**40**), urolithin B (**42**), and 8-*O*-methylurolithin A (**50**) also showed antiproliferative effect on human bladder cancer T24 cells [82].

### 4.3. Phytotoxicity

The metabolites from fungal pathogens are usually toxic to plants and are called phytotoxins which are divided into host-specific [83,84] and host non-specific toxins [85,86]. Some *Alternaria*-derived dibenzo-α-pyrones were approved as the host-specific phytotoxins including altenuene (**1**), alternariol (**10**), alternariol 9-methyl ether (**11**), alternuisol (**15**), and dehydroaltenusin (**17**) [9,10,37,39,41,45].

### 4.4. Antioxidant Activity

Urolithin A (**40**), isourolithin A (**41**), and urolithin B (**42**) from the fruits of *Trapa natans* showed antioxidant activity. Among them, isourolithin A (**41**) showed the strongest, and urolithin B (**42**) showed weak antioxidative effect [4]. As ellagic acid and ellagitannins are extremely poorly absorbed in gut, urolithins appear to be responsible for biological activities related to the intake of ellagitannins. Most of urolithins (*i.e.*, urolithins A, C, and D) exhibited antioxidant activity in a cell-based assay [6]. However, there have been contradictory reports on their antioxidant capacity [62,87]. Recently, urolithins were revealed to display both antioxidant and pro-oxidant activities depending on assay system and conditions by using oxygen radical absorbance capacity (ORAC) assay, three cell-based assays, copper-initiated pro-oxidant activity (CIPA) assay, and cyclic voltammetry. Urolithins were screened to be the strong antioxidants in the ORAC assay, but mostly pro-oxidants in cell-based assays [88]. The antioxidant activity of urolithins is very likely mediated exclusively by the hydrogen atom transfer (HAT) mechanism. The hydrogen atom is donated by the phenolic hydroxyl group [88].

### 4.5. Antiallergic Activity

Urolithin A (**40**), isourolithin A (**41**), and urolithin B (**42**), from the feces of *Trogopterus xanthipes* showed hyaluronidase inhibitory activities with IC_50_ values of 1.33, 1.07 and 2.33 mM, respectively that indicated their antiallergic activity [11]. TMC-264 (**28**) from the fungus *Phoma* sp. TC 1674 [47] selectively inhibited tyrosine phosphorylation of STAT6, and also inhibited the complex formation of phosphorylated STAT6 and its recognition sequence. Therefore, TMC-264 (**28**) would inhibit IL-4 signaling and would be useful in the treatment of allergic disease [47,89,90].

### 4.6. Other Bioactivities

Other biological activities of dibenzo-α-pyrones include antimicrobial, antimalarial, antinematodal activities as well as calmodulin-dependent, estrogenic and antiestrogenic, and acetylcholinesterase (AChE) inhibitory activities. Alternariol 9-methyl ether (**11**) from endophytic *Alternaria* spp. exhibited inhibitory activity on the appressorium formation of *Magnaporthe grisea* (*M. oryzae*) with IC_50_ value of 51.0 μg/mL [34]. Alternariol 9-methyl ether (**11**), botrallin (**16**) and palmariol B (**27**) from endophytic fungus *Hyalodendriella* sp. Ponipodef12 showed moderate antimicrobial activity [12,43].

2,3,4,9,10-Pentahydroxy-6*H*-dibenzo[*b*,*d*]pyran-6-one (**37**) from the plants *Chrozophora senegalensis* (Euphorbiaceae) and *Sebastiania chamaelea* (Euphorbiaceae) showed moderate antimalarial activity (IC_50_ > 10 μg/mL) on *Plasmodium falciparum* [56]. Alternariol 9-methyl ether (**11**), botrallin (**16**) and palmariol B (**27**) from endophytic fungus *Hyalodendriella* sp. Ponipodef12 also showed moderate antinematodal inhibitory activity on *Caenorhabditis elegans* [12].

Dehydroaltenusin (**17**) from *Penicillium verruculosum* IAM-13756 inhibited the calmodulin-dependent activity of myosin light chain kinase (MLCK) with IC_50_ value of 0.69 μM [46].

Both urolithins A (**40**) and B (**42**) from human feces exhibited estrogenic and antiestrogenic activities, which suggested that consumption of ellagitannin-containing foodstuffs such as pomegranate, walnuts, berries, and oak-aged wines may exert some proestrogenic/antiestrogenic effects [91].

Alternariol 9-methyl ether (**11**), botrallin (**16**) and palmariol B (**27**) from endophytic fungus *Hyalodendriella* sp. Ponipodef12 showed moderate AChE inhibitory activity [12]. Botrallin (**16**) from the endophytic fungus *Microsphaeropsis olivacea* was also screened to have AChE inhibitory activity with IC_50_ value as 6.1 μg/mL [13].

## 5. Conclusions and Future Perspectives

We have just clarified one part of the dibenzo-α-pyrones from fungi, plants and bacteria. The remaining dibenzo-α-pyrones in bioorganisms need to be further identified. In recent years, more and more dibenzo-α-pyrones have been isolated from plant endophytic fungi. These endophytic fungi could be the rich sources of biologically active compounds that are indispensable for medicinal and agricultural applications [92,93]. In most cases, biological activities, structure-activity relationships, and modes of action of dibenzo-α-pyrones were only primarily investigated.

The potential applications of dibenzo-α-pyrones as antitumor agents, antiallergics, antioxidants, and antimicrobials have attracted considerable interest within the pharmaceutical community. Chemical syntheses have been achieved for a few bioactive dibenzo-α-pyrones such as altenuene (**1**) [94], isoaltenuene (**2**) [94], neoaltenuene (**5**) [95], alternariol (**10**) [96], alternariol 9-methyl ether (**11**) [96], altertenuol (**15**) [97], dehydroaltenusin (**17**) [98], graphislactones A (**18**), C (**20**), D (**21**) and H (**25**) [99], TMC-264 (**28**) [90], lysilactone A (**34**) [5], urolithins A-C (**40**, **42** and **43**) [100], and urolithin M-7 (**48**) [101]. In addition, some dibenzo-α-pyrones are the important precursors of many synthetic drugs [14,15,16,17,18].

With comprehensive understanding of the biosynthetic pathways of some dibenzo-α-pyrones in the next few years, we may be able to effectively not only increase the yields of bioactive dibenzo-α-pyrones, but also block the biosynthesis of some toxic dibenzo-α-pyrones (*i.e*., phytotoxins and mycotoxins) [1].
